# Perspectives from Hysterectomy Specimens on the Hidden Malignancy Risk in HSIL Patients with Surgical Margin Continuity

**DOI:** 10.3390/medicina62010077

**Published:** 2025-12-30

**Authors:** Gökşen Görgülü, Muzaffer Sanci

**Affiliations:** 1 Department of Gynecologic Oncology, Gaziantep City Hospital, 27470 Gaziantep, Turkey; 2 Department of Gynecologic Oncology, Izmir City Hospital, 35540 Izmir, Turkey

**Keywords:** loop electrosurgical excision procedure, cold-knife conisation, adenocarcinoma in situ, squamous cell carcinoma

## Abstract

*Background and Objectives:* We aim to examine the histopathological results following hysterectomy performed due to insufficient cervical tissue in patients diagnosed with high-grade squamous intraepithelial lesions (HSILs) who underwent the loop electrosurgical excision procedure (LEEP) and cold-knife conisation (CKC) and exhibited continuity at the surgical margin and residual disease. *Materials and Methods:* Thirty-four patients who underwent hysterectomy due to insufficient cervical tissue and had HSILs at the surgical margin were included in this study. The following information was analysed: age, body mass index (BMI), parity, menopausal status (premenopausal/postmenopausal), smoking history, smear result, HPV result, colposcopic cervical biopsy result, transformation zone information, LEEP+Endocervical Curettage (ECC) histopathological result, CKC+ECC histopathological result, hysterectomy material histopathological result, presence or absence of cervical glandular involvement, and presence or absence of residual lesions in the hysterectomy material. *Results:* The mean (±SD) age of the study cohort was 46.7 ± 8.3 years, the mean BMI was 27.4 ± 2.3 kg/m^2^, and the mean parity was 2.5 ± 0.7. According to the results of the hysterectomy performed on these 34 patients, in whom Cervical Intraepithelial Neoplasia 3 (CIN3) continuity at the surgical margin and the inability to perform re-excision were determined, 8 patients (23.5%) had CIN2, 19 patients (55.9%) had CIN3, 3 patients (8.8%) had adenocarcinoma in situ, and 4 patients (11.8%) had squamous cell carcinoma (SCC). Histopathological examinations of the hysterectomy specimens revealed negative surgical margins in all patients, while glandular involvement was present in 13 patients (34.2%). *Conclusions:* It should be borne in mind that patients with HSILs showing continuity at the surgical margin may have an underlying squamous cell carcinoma. These patients should be carefully evaluated for hysterectomy if they do not have sufficient cervical tissue for repeat excisional procedures.

## 1. Introduction

Cervical squamous intraepithelial lesions are classified into two categories: low-grade squamous intraepithelial lesions/Cervical Intraepithelial Neoplasia 1 (LSILs/CIN1) and high-grade squamous intraepithelial lesions/Cervical Intraepithelial Neoplasia 2-3 (HSILs/CIN2-3) [1]. Since HSILs carry an estimated 5–22% risk of progression to invasive cervical carcinoma, therapeutic intervention is necessary [2].

The aim of treatment for patients with HSILs is to completely remove the lesion, ensure that any underlying cervical cancer is not missed, and prevent the lesion from progressing to cervical cancer. The treatments used are cold-knife conisation (CKC) and the loop electrosurgical excision procedure (LEEP) [3]. After these procedures, residual lesions are observed in 20–25% of patients [4]. Patients with residual lesions are at risk of recurrent HSILs and invasive cancer; therefore, these patients must be efficiently identified and managed [5].

Hysterectomy is not the first choice of treatment for patients with HSILs or following excisional procedures [6], but it may become necessary in cases of persistent HSILs where re-excision is technically impossible or where the patient has accompanying benign gynaecological indications (symptomatic myoma, adenomyosis, uterine prolapse, etc.) [7,8]. Our aim in this study is to examine the histopathological results following hysterectomy performed due to insufficient cervical tissue in patients diagnosed with HSILs who underwent LEEP and CKC and had residual disease with continuity at the surgical margin.

## 2. Materials and Methods

**Figure 1:**
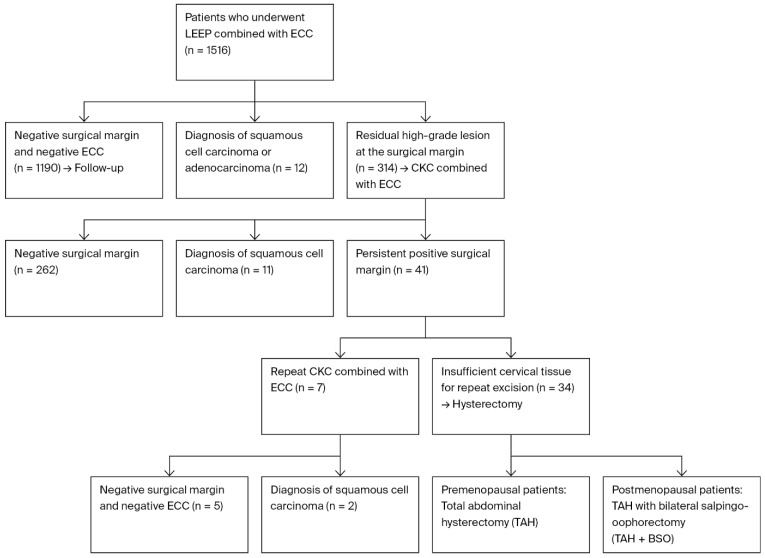
Flow diagram showing patient selection from initial LEEP to hysterectomy inclusion.

The included patients were selected from the 1516 patients who underwent the loop electrosurgical excision procedure and endocervical canal curettage (LEEP+ECC) at our clinic between January 2015 and January 2023, following a colposcopic biopsy result showing an HSIL. Of the 1516 patients who underwent LEEP+ECC, 1190 had negative surgical margins and ECC results and were followed up with, while squamous cell carcinoma and adenocarcinoma were detected in 12 patients. In 314 patients, CKC+ECC was performed due to the persistence of lesions at the surgical margin. Surgical margin positivity was defined as the presence of an HSIL (CIN2-3) at the surgical margin. Of these 314 patients, 262 had negative surgical margins, and squamous cell carcinoma was detected in 11 patients. In 41 patients, another conisation procedure was performed due to the persistence of the lesion at the surgical margin. CKC+ECC was performed again in 7 of the 41 patients; the surgical margin and ECC results were negative in 5 patients, while squamous cell carcinoma (SCC) was encountered in 2 patients. For the other 34 patients, a decision was made to perform a hysterectomy because the gynaecological examination revealed insufficient cervical tissue for repeat excision. The adequacy of cervical tissue was determined by visual examination using a speculum and by bimanual palpation of the cervix and vaginal examination. A total abdominal hysterectomy was performed in premenopausal patients, whereas postmenopausal patients underwent a total abdominal hysterectomy combined with bilateral salpingo-oophorectomy (Figure 1). Based on the specified criteria, a total of 34 patients were included in the study. Ethical approval was obtained from the Ethics Committee of the İzmir Tepecik Training and Research Hospital, University of Health Sciences (approval no. 2023/10-18; date: 20 November 2023), prior to the initiation of this study. This study was carried out retrospectively in compliance with the Declaration of Helsinki and the Good Clinical Practice principles, and all medical data were retrieved from patient medical records.

The following patient information was collected: age, BMI, parity, menopausal status (premenopausal/postmenopausal), smoking history, Pap smear and HPV results at the initial examination, colposcopic cervical biopsy results, transformation zone information, LEEP+ECC histopathological results, CKC+ECC histopathological results, hysterectomy material histopathological results, presence or absence of cervical glandular involvement, and presence or absence of residual lesions in the hysterectomy material.

The LEEP and CKC procedures were performed by gynaecological oncologists in the lithotomy position under anaesthesia and sterile conditions. LEEP was performed in a single pass from left to right using a loop electrode with 50 W cutting and 30 W coagulation currents, followed by ECC. The procedure was concluded by controlling bleeding with a bulbous electrode. The CKC procedure was performed by inserting a hysterometer into the cervical canal to determine the endocervical dimensions and by placing haemostatic sutures laterally on both sides of the cervix (corresponding to approximately the 3 and 9 o’clock directions) using a number 11 bistoury with an angled handle. ECC was then performed again, and the procedure was concluded after controlling the bleeding.

All materials sent to pathology were marked with a guiding suture placed superiorly at the cervical 12 o’clock direction. The entire endocervical and ectocervical margins were examined by specialist gynaecologic pathologists to determine the presence of residual lesions. Following macroscopic evaluation, the lesions were processed via paraffin embedding, sectioning, and staining with haematoxylin and eosin. The specimens were then examined, and the surgical margin involvement and presence of residual disease were recorded.

### Statistical Analysis

Data analysis was performed utilising SPSS Statistics (v22.0; IBM Corp., Chicago, IL, USA). The continuous variables (age, BMI, and parity) are reported as the mean ± standard deviation (SD). The distribution of continuous variables was tested using the Shapiro–Wilk test. Age and BMI were normally distributed and, therefore, presented as mean ± standard deviation (SD). Parity did not follow a normal distribution; however, as it was used descriptively, it was retained in its original format. The Wilson method, which yields more precise estimates for small samples, was used to compute 95% confidence intervals (CIs) for proportions and to summarise categorical variables as frequencies and percentages (n, %).

To prevent overfitting and unstable estimates, no regression or inferential hypothesis testing (such as χ^2^ or logistic regression) was carried out due to the small number of malignant outcomes (adenocarcinoma in situ or SCC, n = 7) and the small number of patients (n = 34). Rather, a precision-based descriptive method was used, with a focus on presenting 95% CIs to show how reliable the proportions are.

All results were interpreted descriptively; no *p*-value threshold was used.

## 3. Results

The mean (±SD) age of the study cohort was 46.7 ± 8.3 years, the mean BMI was 27.4 ± 2.3 kg/m^2^, and the mean parity was 2.5 ± 0.7. A total of 17 patients (50%; 95% CI: 34.1–65.9) were premenopausal, 17 were postmenopausal, and 21 (61.8%; 95% CI: 45.0–76.1) had a history of smoking. When the physical examination findings were examined, 13 patients (38.2%; 95% CI: 23.9–55.0) were found to have type 1 transformation zones, 17 patients (50%; 95% CI: 34.1–65.9) had type 2, and 4 patients (11.8%; 95% CI: 4.7–26.6) had type 3 (Table 1).

Examination of the smear results from the patients’ initial examinations found that 21 (61.8%; 95% CI: 45.0–76.1) had normal smear results, 8 (23.5%; 95 CI: 12.4–40.0) had atypical squamous cells of undetermined significance (ASC-US), and 5 (14.7%; 95% CI: 6.4–30.1) had atypical squamous cells—we also cannot exclude high-grade squamous intraepithelial lesions (ASC-H). The detection of Human Papilloma Virus (HPV) in the initial examination found HPV 16 in 24 patients (70.6%; 95% CI: 53.8–83.2), HPV 18 in 4 patients (11.8%; 95% CI: 4.7–26.6), HPV 33 in 2 patients (5.9%; 95% CI: 1.6–19.1), HPV 45 in 2 patients (5.9%; 95% CI: 1.6–19.1), and HPV 56 in 1 patient (2.9%; 95% CI: 0.5–14.9). In one patient (2.9%; 95% CI, 0.5–14.9), HPV 16 and 45 were both detected (Table 1). In the colposcopic biopsy results, CIN2 was found in seven patients (20.6%; 95% CI: 10.4–36.2), while CIN3 was detected in 27 patients (79.4%; 95% CI: 63.8–89.6) (Table 2).

LEEP+ECC was performed on all patients, and CIN3 was detected in all of them. CIN3 continuity was observed at the ectocervical margin in 23 patients (67.6%; 95% CI: 50.8–80.9) and at both the endocervical and ectocervical margins in 11 patients (32.4%; 95% CI: 19.1–49.2). The subsequent CKC+ECC results in these patients showed CIN3 continuity at the ectocervical margin in 21 patients (61.8%; 95% CI: 45.0–76.1) and at both the endocervical and ectocervical margins in 13 patients (38.2%; 95% CI: 23.9–55.0). Using the hysterectomy results of these 34 patients, CIN3 continuity at the surgical margin and whether re-excision could be performed were determined. A total of 8 patients (23.5%; 95% CI: 12.4–40.0) had CIN2, 19 patients (55.9%; 95% CI: 39.5–71.1) had CIN3, 3 patients (8.8%; 95% CI: 3.0–23.0) had adenocarcinoma in situ (AIS), and 4 patients (11.8%; 95% CI: 4.7–26.6) had squamous cell carcinoma (SCC). Histopathological examination of the hysterectomy specimens revealed negative surgical margins in all patients, while glandular involvement was present in 13 patients (38.2%; 95% CI: 23.9–55.0) (Table 2).

When examining the characteristics of the seven patients who underwent hysterectomy and whose results were reported as SCC and adenocarcinoma in situ, it was observed that the age range was between 36 and 49, one patient had no history of smoking, and adenocarcinoma in situ was detected in this patient. In the four patients with SCC, three had HPV 16, while one had both HPV 16 and 45. According to the smear results, three of these four patients had HSILs, while one had a normal smear, and the colposcopic biopsy results showed CIN3 in all cases. Regarding glandular involvement, only one patient had no glandular involvement, and all patients were diagnosed with stage IA1 cervical cancer and, therefore, did not receive adjuvant treatment. No recurrence was observed during follow-up. In the three patients reported to have adenocarcinoma in situ, two patients had HPV 18, while one patient had HPV 16. Two patients had normal smear results, one patient had ASC-US, and all three patients were found to have CIN3 based on colposcopic biopsy results. Only one patient had no glandular involvement (Table 3).

## 4. Discussion

In our study, the post-hysterectomy histopathological results of patients who demonstrated continuity at the surgical margins and had insufficient remaining cervical tissue to justify a repeat excisional procedure were thoroughly examined; in this group of patients, the presence of an underlying or occult squamous cell carcinoma was found to be a possible and clinically relevant finding.

Surgical margin positivity observed after LEEP and CKC is a very important risk factor for invasive lesions [9]. There are several reasons for the continuity of the lesion at the surgical margin after LEEP and CKC. These include lesion-related factors (extensive spread and glandular involvement), patient-related factors (advanced age; atrophic cervix; type 3 transformation zone; postmenopause; and high-risk factors, particularly HPV16/18 persistence), and surgeon-related factors (insufficient excision depth and volume) [5,6,10]. In our study, the mean age of the patients was 46.7 ± 8.3 years, 4 patients (11.8%) had type 3 transformation zones, 17 patients (50%) were postmenopausal, 24 patients (70.6%) had HPV16, 4 patients (11.8%) had HPV18, 1 patient (2.9%) had HPV 16 and 45 co-infection, and 13 patients (38.2%) had glandular involvement.

Hysterectomy is not the primary treatment for HSILs [11,12]; however, studies have shown that there are situations where hysterectomy is required [13,14]. In a multicentre study where hysterectomy was performed on 314 patients due to CIN, the histopathological results revealed cervical cancer in 25 patients (7.9%); 9 patients were stage IA1, 6 patients were stage IA2, and 10 patients were stage IB1. Adjuvant treatment was administered to eight of these patients [15]. In another multicentre study involving 242 patients who underwent hysterectomy due to CIN, cervical cancer was encountered in 42 patients (17.3%) [16]. In our study, squamous cell carcinoma was observed in four patients (11.8%), which is consistent with the rates in the literature. These four patients with SCC were classified as stage IA1 and did not receive adjuvant therapy.

Comprehensive multivariate analyses in previous studies have shown residual lesions and malignant pathologies in hysterectomy specimens to be significantly associated with several independent risk factors, including positive surgical margins, postmenopausal status, HPV 16/18 positivity, and glandular involvement [17,18,19]. In our study, particularly in the AIS and SCC cases, only three patients were premenopausal, and two patients did not have glandular involvement. Positive surgical margins were present in all patients prior to hysterectomy, and HPV 16/18 positivity was also present in all patients. However, HPV 16 positivity was present in all SCC cases, and HPV 18 was detected in two out of three AIS cases. This is consistent with the literature, which indicates that HPV 16 is more common in squamous lesions, and HPV 18 plays a dominant role in cervical adenocarcinomas [20]. Another important point in our study is that AIS and SCC were observed in HSIL or hysterectomy materials after colposcopy in patients with normal smear results. This situation highlights the low sensitivity of cervical cytology and the necessity of HPV testing in conjunction with cytology [21].

Some studies have reported that the glandular spread of HSIL is insufficient for predicting glandular neoplasia; however, the presence of deep endocervical crypts and the type 3 transformation zone increases oncological risk and the possibility of incomplete surgery [22,23,24]. In our study, glandular involvement was detected in 13 patients (38.2%) in hysterectomy specimens, while AIS was detected in only three patients (8.8%). This result suggests that glandular involvement may be a less aggressive precursor lesion and that some patients with glandular involvement progress to AIS. Therefore, the presence of glandular involvement may require closer follow-up and repeated excision, even if AIS is not confirmed.

We deliberately chose not to perform inferential or comparative statistical analyses, as the number of adverse outcomes in our sample was too small to produce statistically stable or clinically meaningful results. Conducting such tests under these limited conditions could have resulted in unreliable parameter estimates and an increased likelihood of committing type II statistical errors. Instead, a descriptive method that depended on accuracy was used, and Wilson’s 95% confidence intervals were used to show how accurate and reliable the observed proportions were. The Wilson score interval offers enhanced coverage probabilities for small samples or proportions approaching zero or one, in contrast to the traditional Wald interval [25,26]. This method has been recommended for retrospective studies with small sample sizes as it improves the interpretability of the findings and helps prevent misleading impressions of statistical significance [27].

One of the key advantages of our study is that it addresses a clinically common but under-researched topic in the literature, and it is the first study to examine the outcomes of hysterectomy following repeated excisional procedures. Its weaknesses are that it is retrospective and has a small sample size. Furthermore, as there is no information on the volume and depth of the excision in LEEP and CKC, surgical factors could not be fully analysed. For these reasons, we emphasise that our findings should be validated through well-designed, large-scale prospective studies to ensure greater reliability and generalisability of the results.

## 5. Conclusions

The possibility of an underlying squamous cell carcinoma should be considered in patients diagnosed with HSILs showing continuity at the surgical margin. These patients should be carefully evaluated for hysterectomy if they do not have sufficient cervical tissue for repeat excisional procedures.

## Figures and Tables

**Table 1:** Demographic and clinical and histopathological characteristics of patients.

Parameter	N (%) or Mean ± SD	95% CI
Age	46.7 ± 8.3	-
BMI (kg/m^2^)	27.4 ± 2.3	-
Parity	2.5 ± 0.7	-
Menopausal Status		
Premenopausal	17 (50)	34.1–65.9
Postmenopausal	17 (50)	34.1–65.9
Smoking History		
Yes	21 (61.8)	45.0–76.1
No	13 (38.2)	23.9–55.0
Transformation Zone Type		
Type 1	13 (38.2)	23.9–55.0
Type 2	17 (50)	34.1–65.9
Type 3	4 (11.8)	4.7–26.6
Cytology (Pap Smear) Findings		
Normal	21 (61.8)	45.0–76.1
ASC-US	8 (23.5)	12.4–40.0
ASC-H	5 (14.7)	6.4–30.1
AGUS	0 (0)	-
LSIL	0 (0)	-
HSIL	0 (0)	-
SCC	0 (0)	-
HPV Genotype		
Type 16	24 (70.6)	53.8–83.2
Type 18	4 (11.8)	4.7–26.6
Type 33	2 (5.9)	1.6–19.1
Type 45	2 (5.9)	1.6–19.1
Type 56	1 (2.9)	0.5–14.9
Types 16 and 45	1 (2.9)	0.5–14.9

HSIL, high-grade squamous intraepithelial lesion; SCC, squamous cell carcinoma; HPV, human papillomavirus; ASC-US, atypical squamous cells of undetermined significance; ASC-H, atypical squamous cells—cannot exclude HSIL; LSIL, low-grade squamous intraepithelial lesion; CI, confidence interval; SD, standard deviation.

**Table 2:** Histopathological characteristics of patients.

Parameter	N (%)	95% CI
Colposcopic Biopsy Result		
CIN2	7 (20.6)	10.4–36.2
CIN3	27 (79.4)	63.8–89.6
LEEP + ECC Histopathology Result (All CIN3)		
Ectocervical Margin Positive	23 (67.6)	50.8–80.9
Endocervical Margin Positive	0 (0)	-
Combine Margin Positive	11 (32.4)	19.1–49.2
Negative	0 (0)	-
Post-LEEP CKC + ECC Histopathology Result (All CIN3)		
Ectocervical Margin Positive	21 (61.8)	45.0–76.1
Endocervical Margin Positive	0 (0)	-
Combine Margin Positive	13 (38.2)	23.9–55.0
Negative	0 (0)	-
Hysterectomy Histopathology		
CIN2	8 (23.5)	12.4–40.0
CIN3	19 (55.9)	39.5–71.1
Adenocarcinoma In Situ	3 (8.8)	3.0–23.0
Squamous Cell Carcinoma	4 (11.8)	4.7–26.6
Glandular Involvement in Hysterectomy Specimens		
Present	13 (38.2)	23.9–55.0
Absent	21 (61.8)	45.0–76.1
Surgical Margin Status in Hysterectomy Specimens		
Negative	34 (100)	-
Positive	0 (0)	

CIN, cervical intraepithelial neoplasia; LEEP, loop electrosurgical excision procedure; CKC, cold knife conisation; ECC, endocervical curettage; CI, confidence interval; SD, standard deviation.

**Table 3:** Characteristics of patients with histopathology showing squamous cell carcinoma and adenocarcinoma in situ following hysterectomy.

Patient No.	Age	Smoking Status	HPV Type	Smear	Colposcopic Biopsy Histopathology	Hysterectomy Histopathology	Glandular Involvement
1	41	Positive	16	HSIL	CIN3	SCC	Positive
2	36	Positive	16, 45	Normal	CIN3	SCC	Positive
3	46	Positive	16	HSIL	CIN3	SCC	Positive
4	39	Positive	16	HSIL	CIN3	SCC	Negative
5	47	Negative	18	Normal	CIN3	AIS	Positive
6	49	Positive	18	Normal	CIN3	AIS	Positive
7	46	Positive	16	ASC-US	CIN3	AIS	Negative

AIS, adenocarcinoma in situ; SCC, squamous cell carcinoma; HPV, human papillomavirus; CIN, cervical intraepithelial neoplasia; HSIL, high-grade squamous intraepithelial lesion.

## Data Availability

The data supporting the findings of this study are available from the corresponding author upon reasonable request.
